# Scale of dyspnea in COPD: User friendly?

**DOI:** 10.4103/1817-1737.58963

**Published:** 2010

**Authors:** Viroj Wiwanitkit

**Affiliations:** *Wiwanitkit House, Bangkhae, Bangkok, Thailand 10160. E-mail: wviroj@yahoo.com*

Sir,

I read the report on evaluation of scaling system for COPD by Chhabra *et al.*, with a great interest.[1] Chhabra *et al.*, reported that MMRC had poorer relationship to parameters of physiological impairment when compared to BDI and OCD scales. I agree with this finding, however, there is an interesting topic to be discussed. It is accepted that MMRC grade has the most simplicity and it is easy to use. This kind of scale requires less instruments for assessment compared to other two scales. MMRC can be used by general practitioners. To use in a real life, due to the user-friendly nature, it is still the acceptable first choice for rapid screening, isn't it?
